# Tobacco advertising, promotion, sponsorship and youth smoking behavior: The Indonesian 2019 Global Youth Tobacco Survey (GYTS)

**DOI:** 10.18332/tid/174644

**Published:** 2023-12-12

**Authors:** Hario Megatsari, Erni Astutik, Karin Gandeswari, Susy K. Sebayang, Siti R. Nadhiroh, Santi Martini

**Affiliations:** 1 Department of Health Promotion and Behavior Sciences, Faculty of Public Health, Universitas Airlangga, Surabaya, Indonesia; 2 Research Group Tobacco Control, Universitas Airlangga, Surabaya, Indonesia; 3 Department of Epidemiology, Faculty of Public Health, Universitas Airlangga, Surabaya, Indonesia; 4 Research Group for Health and Well-being of Women and Children, Universitas Airlangga, Surabaya, Indonesia; 5 Faculty of Public Health, Universitas Airlangga, Surabaya, Indonesia; 6 School of Health and Life Sciences, Universitas Airlangga, Surabaya, Indonesia; 7 Department of Nutrition, Faculty of Public, University of Airlangga, Surabaya, Indonesia

**Keywords:** tobacco control, youth, TAPS, public health, smoking behavior

## Abstract

**INTRODUCTION:**

Tobacco advertisement, promotion, and sponsorship (TAPS) in all forms influences youth smoking behaviors. TAPS exposure enhances their smoking frequency and vulnerability. A 2018 Indonesia Ministry of Health (MoH) Survey showed increased smoking prevalence among youth aged 10–18 years. Thus, our objective was to analyze the relationship between TAPS and the increased Indonesian youth smoking behavior.

**METHODS:**

We conducted a secondary analysis of the Indonesian 2019 Global Youth Tobacco Survey. The sample size differed in each variable: current smokers n=3386, ever smoker n=3666, and cigarette consumption per day n=1355. We adjusted for socioeconomic and demographic variables and used logistic regression with youth smoking prevalence as the outcome and TAPS variables as the primary exposures.

**RESULTS:**

The current male youth smoker prevalence was 38.3%, ever smoker was 67%, and high consumption per day smoker (≥2 cigarettes per day) was 39.1%. Youth respondents exposed to the promotion or sponsorship of cigarette products showed an increase in three smoking behaviors. In particular, when youth respondents were exposed to more than one type of cigarette promotion (AOR=1.67, 95% CI: 1.33–2.09) or noticed one type of cigarette sponsorship (AOR=2.06, 95% CI: 1.44–2.93), their odds of all three smoking behaviors (current smoker, ever smoker, and high consumption smoker) increased.

**CONCLUSIONS:**

TAPS increase smoking behaviors among Indonesian youth. Therefore, to protect Indonesian youth health in the future, strategic action is needed to reduce youth smoking by banning all forms of TAPS in Indonesia.

## INTRODUCTION

In 2019, approximately 1 billion people worldwide used tobacco products, including 847 million men and 153 million women^1^. A total of 25 million of these tobacco users were teenagers aged 13–15 years^2^. The Southeast Asia Region (SEARO) and Western Pacific Region (WPRO) have the most smokers, accounting for approximately 6.4 million and 4.7 million, respectively^2^. Indonesia is a major contributor to the region’s number of smokers^2^.

Tobacco advertisement, promotion, and sponsorship (TAPS) increase youth frequency and susceptibility to smoking. The World Health Organization (WHO) includes banning TAPS as part of a global tobacco control strategy called MPOWER^3^. WHO described MPOWER as: M– monitoring tobacco use; P– protecting people from tobacco smoke; O– offering help to quit tobacco use; W– warning about the dangers of tobacco; E– enforcing a ban on TAPS; and R– raising taxes on tobacco product^4^. Several researchers have confirmed the association between TAPS and youth smoking behavior^5-8^.

An Indonesia Ministry of Health (IMoH) survey showed increased smoking prevalence among youth aged 10–18 years, from 7.2% in 2013 to 9.1% in 2018^9^. The Indonesian 2019 Global Youth Tobacco Survey (GYTS) found smoking prevalence even higher at 19.2%^10^. This was far from the government’s 2019 target to reduce smoking among children and adolescents to 5.4%^11^. Since 2018, Ministry of Health Republic Indonesia has been processing the amendment on the National Regulation No. 109 of year 2012 (PP 109/2012)^12^. One of the issues that addressed in the amendment version was about banning the TAPS. However, the process of the amendment was not smooth, so that it was cancelled in 2021^13^.

Several previous regional studies have found a relationship between youth smoking behavior and TAPS^14-17^. However, few utilized secondary data to investigate youth smoking behavior and TAPS nationally. This secondary data analysis will benefit the government by providing evidence for adjusting public health policy. Therefore, our objective was to analyze the relationship between TAPS and the increase in Indonesian youth smoking behavior based on the Indonesian 2019 GYTS.

## METHODS

### Data source

We performed a secondary dataset analysis of the Indonesian 2019 GYTS carried out by the Indonesian Ministry of Health in collaboration with WHO and the US Centers for Disease Control and Prevention (CDC). It was conducted in Java, Sumatra, and other regions. GYTS is a cross-sectional survey conducted to monitor tobacco use among school-age (13–17 years) children in public and private schools. The survey was a two-stage sample: The first stage was selecting schools with probability proportional to size (PPS). The second stage was selecting classes at random within schools^18^. Finally, all students from the selected classes were surveyed. We used complete cases for all analyses. The sample size for the analyses of this study differed for each variable (current smokers n=3386, ever smokers n=3666, and smokers with reported cigarette consumption per day n=1355).

### Variables

*Outcome variables*

Our outcome of interest was smoking behavior measured using three variables: current smoker, ever smoker, and cigarette consumption per day. Current smokers were measured using the question: ‘During the past 30 days, on how many days did you smoke cigarettes?’. Respondents who answered zero days were categorized as non-smokers, and respondents who answered ≥1 day were categorized as current smokers. Ever smokers were measured using the yes/no question: ‘Have you ever tried or experimented with cigarette smoking, even one or two puffs?’^18^.

Finally, cigarette consumption per day was measured using the question: ‘Please think about the days you smoked cigarettes (including smoking white cigarettes, hand-rolled cigarettes, or clove cigarettes) during the past 30 days. How many cigarettes did you usually smoke per day?’^19^. Respondents who answered ≤1 cigarette per day were categorized as low cigarette per day smokers. Respondents who answered 2–5 or more cigarettes per day were categorized as high cigarette per day smokers.

*Exposures*

We had three exposures, exposure to tobacco advertisements, tobacco promotions, and tobacco sponsorship. Each independent variable was constructed from questions in the Indonesian 2019 GYTS. Details explaining the construction of the three exposures are included in Supplementary file Table 1.

*Covariates*

Our covariates were sex (female, male), grade (junior high school, senior high school), weekly spending money (in IDR) (>50000, 41000–50000, 31000–40000, 21000–30000, 11000–20000, <11000, or usually do not have any spending money)(1 million IDR about 64 US$)^18^. We also included secondhand smoking, noticed anti-tobacco advertising, and attitude towards smoking behavior as covariates.

Secondhand smoking consisted of three variables: people who smoked inside your home in your presence in the past seven days (on 0, 1–2, 3–4, 5–6, and 7 days); saw anyone who smoked inside the school building or outside school property (yes, no), and do your parents smoke (yes, no). Noticed anti-tobacco advertising had three categories: none, 1, and >1.

The respondents’ attitude toward smoking behavior was assessed by 12 questions:

- Do you think the smoke from other people’s cigarettes smoking is harmful to you?
- Are you in favor of banning smoking inside enclosed public places (such as in health facilities, public transportation, teaching and learning places, shops, restaurants, shopping centers, theaters, cafes, indoor sports clubs)?
- Are you in favor of banning smoking at outdoor public places (such as playgrounds, roadside, building entrances, parks, on the beach, on sports fields)?
- Do you think the price of cigarettes should be increased?
- Do you think the sale of cigarette sticks should be banned?
- Do you think cigarette advertising should be banned?
- If one of your best friends offered you a cigarette product, would you use it?
- Once someone has started smoking tobacco, do you think it would be difficult for them to quit?
- Do you think smoking tobacco helps people feel more comfortable or less comfortable at celebrations, parties, or in other social gatherings?
- Do you agree or disagree with the following: ‘I think I might enjoy smoking a cigarette.’?^20^
- Do you think smoking cigarettes is harmful to your health?
- Do you think it is safe to smoke tobacco for only a year or two as long as you quit after that?^19^

Each attitude question was categorized into a zero (0) or one (1), where students’ responses that indicated a risky smoking attitude received a higher score and vice versa. Finally, the respondents’ scores were summed into a total score and based on the median, categorized into positive attitudes (≤3) and negative attitudes (≥4).

### Statistical analysis

We used complete cases and weighted for the complex survey sampling design. Bivariable analysis was conducted to analyze the crude odds ratio to measure the variable associations. We determined the relationship between the outcomes and independent variables using multivariable logistic regression and adjusted for grade, weekly spending money, secondhand smoking, noticed anti-tobacco advertising and attitude towards smoking behavior. Because very few females were smokers, we only analyzed male smokers in the logistic regressions. Odd ratio (OR) and adjusted odd ratio (AOR) with 95% CI were used for the logistic regression analysis. The data were analyzed using StataCorp. 2019 (Stata Statistical Software: Release 16. College Station, TX: StataCorp LLC).

## RESULTS

In general, our findings can be seen in Figures 1 and 2 in the Supplementary file. For all outcomes, the percentage of males was much greater than females (Supplementary file Table 2). Only 2.3% of females vs 38% of males were current smokers. By education level, current smoker and ever smoker percentages were similar, around 18% and 37%, respectively. Among those in senior high school, cigarette consumption per day was greater, approximately 55%. Weekly spending money varied across the outcome variables. For current smokers, 21.9% spent less than IDR 11000. Among ever smokers, 40% spent IDR 11000–20000. Finally, among respondents who smoked at least one cigarette per day, almost 50% spent within IDR 50000 (Supplementary file Table 2).

In bivariate comparisons, all independent and outcome variables were significantly related. The percentage of the exposure to more than one type of tobacco advertisement for each variable was 20.3% (current smoker), 40% (ever smoker), and 39.2% (cigarette consumption per day). Tobacco promotions exposure to more than one type of tobacco promotion in each variable was 39.9% (current smoker), 58.2% (ever smoker), and 47.5% (cigarette consumption per day). For tobacco sponsorship exposure, the percentages of the exposure of more than 1 type of tobacco sponsorship in each variable were 40.4% (current smoker), 58.7% (ever smoker), and 38.2% (cigarette consumption per day) (Supplementary file Table 2).

Compared to adolescents not exposed, adolescents exposed to more than one tobacco promotion had 1.67 times higher odds of being a current smoker (AOR=1.67; 95% CI: 1.33–2.09). Compared to unexposed adolescents, adolescents exposed to more than one tobacco sponsorship exposure and at least one tobacco sponsorship exposure had 2.06 times (AOR=2.06; 95% CI: 1.44–2.93) and 1.83 (AOR=1.83; 95% CI: 1.45–2.31) times higher odds, respectively, to be smokers (Table 1).

**Table 1:** Determinants of male youth smoking behavior analysis from Indonesia 2019 GYTS

*Outcomes*	*Variables*	*OR*	*S.E.*	*95% CI*	*p*	*AOR*	*S.E.*	*95% CI*	*p*
**Current smoking** (n=3386)	**Tobacco advertisement exposure**								
	None (Ref.)	1				1			
	1	1.46	0.25	1.04–2.05	*	1.13	0.20	0.79–1.60	0.50
	>1	1.76	0.30	1.24–2.48	**	0.96	0.16	0.69–1.35	0.83
	**Tobacco promotion exposure**								
	None (Ref.)	1				1			
	1	1.49	0.15	1.23–1.81	***	1.03	0.13	0.80–1.32	0.83
	>1	3.22	0.34	2.61–3.97	***	1.71***	0.19	1.36–2.14	***
	**Tobacco sponsorship exposure**								
	None (Ref.)	1				1			
	1	2.82	0.28	2.31–3.43	***	1.89***	0.22	1.50–2.38	***
	>1	3.56	0.56	2.61–4.87	***	2.13***	0.37	1.51–3.02	***
**Ever smoking** (n=3666)	**Tobacco advertisement exposure**								
	None (Ref.)	1				1			
	1	1.51	0.22	1.13–2.03	**	1.22	0.20	0.88–1.70	0.23
	>1	1.66	0.28	1.18–2.33	**	0.99	0.19	0.67–1.45	0.94
	**Tobacco promotion exposure**								
	None (Ref.)	1				1			
	1	1.63	0.23	1.23–2.16	**	1.31	0.21	0.96–1.80	0.09
	>1	2.10	0.27	1.62–2.71	***	1.30	0.18	0.98–1.73	0.07
	**Tobacco sponsorship exposure**								
	None (Ref.)	1				1			
	1	1.90	0.21	1.53–2.36	***	1.31*	0.15	1.05–1.64	*
	>1	2.24	0.38	1.60–3.14	***	1.41*	0.24	1.01–1.98	*
**Cigarette consumption per day** (n=1355)	**Tobacco advertisement exposure**								
	None (Ref.)	1				1			
	1	2.73	1.23	1.12–6.69	*	2.75*	1.21	1.14–6.63	*
	>1	3.26	1.20	1.56–6.81	**	2.92**	1.02	1.45–5.87	***
	**Tobacco promotion exposure**								
	None (Ref.)	1				1			
	1	1.24	0.19	0.91–1.70	0.17	1.03	0.18	0.73–1.45	0.86
	>1	1.86	0.27	1.40–2.47	***	1.51*	0.25	1.09–2.11	*
	**Tobacco sponsorship exposure**								
	None (Ref.)	1				1			
	1	1.71	0.25	1.27–2.30	**	1.37	0.23	0.98–1.92	0.06
	>1	1.27	0.17	0.97–1.66	0.08	0.96	0.16	0.69–1.33	0.79

AOR: adjusted odds ratio; adjusted for grade, weekly spending money, secondhand smoking, noticed anti-tobacco advertising, and attitude towards smoking behavior. S.E. standard error.

*** p<0.001,

** p<0.01,

* p<0.05.

Adolescents exposed to tobacco sponsorship had 1.28 (AOR=1.28; 95% CI: 1.02–1.61) times higher odds of having ever smoked. Compared to adolescents not exposed, adolescents exposed to more than one or at least one tobacco advertisement had 2.95 (AOR=2.95; 95% CI: 1.44–6.02) and 2.91 (AOR=2.91; 95% CI: 1.44–6.02) times higher odds to smoke more cigarettes per day, respectively. Finally, compared to adolescents not exposed, adolescents exposed to more than one tobacco promotion in the last 30 days had 1.58 (AOR=1.58; 95% CI: 1.15–2.18) times higher odds of smoking more cigarettes per day (Table 1).

## DISCUSSION

The study showed that adolescents who were exposed to more than one tobacco promotion had 1.67 times the odds of reporting themselves as smokers compared to those who were not. Adolescents exposed to more than one tobacco sponsorship exposure and at least one tobacco sponsorship exposure had 2.06 times and 1.83 times higher odds of being smokers, respectively, than unexposed adolescents. Also, the study found that adolescents who were exposed to tobacco sponsorship had 1.28 times the likelihood of ever smoking. Adolescents who saw at least one or more tobacco advertisements had odds of smoking more cigarettes per day that were 2.95 and 2.91 times higher than those who were not exposed. Finally, teenagers exposed to many tobacco promotions had 1.58 times higher odds of smoking more cigarettes per day than adolescents who were not exposed.

WHO defines two main TAPS types: 1) Tobacco advertising and tobacco promotion, and 2) Tobacco sponsorship. Tobacco advertising and tobacco promotion is ‘any form of commercial communication, recommendation or action with the aim, effect or likely effect of promoting a tobacco product or tobacco use either directly or indirectly.’^20^ Tobacco advertising is promoting tobacco products for example on television, in online media, and outdoor venues. Tobacco promotion examples are giving free cigarette products to youth, and distributing free items (e.g. shirts, pen, backpack, hat) with a tobacco logo on them. Tobacco sponsorship is ‘any event, activity or individual with the aim, effect or likely effect of promoting a tobacco product or tobacco use either directly or indirectly.’^20^, for example at live music events, sporting events, and community events.

We found three significant relationships between TAPS exposures and youth smoking behaviors. Current youth smoking was strongly associated with tobacco promotion and sponsorship. This confirmed previous research. In 2017, an Indonesian study found that 60.6% of teenage smoker respondents were exposed to tobacco promotion at live concerts^5^. Music concerts are one of the many venues used to promote and maintain smoking behavior^21^. Sporting events, such as football competitions or badminton tournaments, are also used by the tobacco industry to promote their products. They will spend large amounts of money to sponsor football, badminton, and other teams. Siahaya and Smits^22^ found that Djarum, one of the three largest Indonesian tobacco companies, used the Sport Corporate Social Responsibility (SCSR) program to promote their products. Unfortunately, In Indonesia, the tobacco industry is not banned from supporting sporting events^23^. Adolescents’ exposure to tobacco sponsorship at various events, such as sport events, music events or community events was associated with past ever smoking.

Media exposure, such as television, online media and in outdoor venues, also increases the odds that youth will smoke more than one cigarette a day. These results are consistent with the majority of other studies that have discovered a connection between children’s and teens’ smoking behavior and exposure to TAPS on television^24^. Television tobacco advertising is crucial to the industry’s strategy to acquire and maintain youth smokers. According to the Indonesian Food and Drugs Administration (FDA), there were about 42597 tobacco-related television commercials in 2020^25^.

A variety of Indonesia institutions, both governmental and civic, have already undertaken tobacco control efforts. In terms of TAPS, since 2012 several institutions and agencies have had efforts to implement an amendment to the Broadcasting Law No. 32 of 2002. In 2017, civic organizations submitted a judicial review on Article 46 Paragraph 3 of the Law regarding cigarette advertisements to the Constitutional Court of the Republic Indonesia. Unfortunately, the judicial review was not successful^26^. The Indonesian Child Protection Commission urged the Indonesian Government to revise National Law No. 32 of 2002, to ban all kinds of TAPS to protect children and teenagers from smoking behavior^27^.

In Indonesia, the social media have grown to be a significant platform for advertising commercial goods. In 2021, 170 million people (or 61.8% of the population) actively utilized social media^28^. Instagram, Facebook, and YouTube are the main online sources of TAPS exposure for Indonesian teenagers^5^. In 2017, the tobacco business invested over 481.3 million US$ in digital advertising. By 2021, this sum will have nearly doubled to 844.9 million US$. Unfortunately, no regulations exist to limit the use of TAPS on the internet and social media, so the tobacco industry can promote its goods on the internet and social media without limit, everyday^23^. Our study provides evidence to support the advocacy process for the amendment of National Law No. 32 of 2002.

### Limitations

Our study has several limitations. Though youth smoking behavior is multifactorial, our choice of variables depended on the contents of the GYTS 2019. Our analysis was also limited to only male smokers, because the number of female smokers was too small. Social desirability bias may have played a role in this; female students may not have wished to reveal their smoking behavior. Additionally, utilizing a cross-sectional approach did not allow for the elucidation of causal connection between TAPS and youth smoking behavior.

## CONCLUSIONS

TAPS exposure increases the risk of smoking behaviors among Indonesian youth. Strategic action is needed to reduce youth smoking by banning all forms of TAPS in Indonesia.

## Data Availability

The data supporting this research are available from the corresponding author on reasonable request.

## References

[1] World Health Organization (2021). WHO report on the Global Tobacco Epidemic, 2021. Addressing new and emerging products.

[2] Lian TY, Dorotheo U (2021). The Tobacco Control Atlas: ASEAN Region.

[3] WHO. World Health Organization (2008). Report on the Global Tobacco Epidemic, 2008: The MPOWER Package.

[4] World Health Organization (2008). MPOWER: a policy package to reverse the tobacco epidemic. WHO report on the global tobacco epidemic.

[5] Septiono W, Kuipers MAG, Ng N, Kunst AE (2022). Self-reported exposure of Indonesian adolescents to online and offline tobacco advertising, promotion and sponsorship (TAPS). Tob Control.

[6] Efendi F, Aidah FN, Has EMM, Lindayani L, Reisenhofer S (2019). Determinants of smoking behavior among young males in rural Indonesia. Int J Adolesc Med Health.

[7] Liang YC, Liao JY, Lee CT, Liu CM (2022). Influence of personal, environmental, and community factors on cigarette smoking in adolescents: a population-based study from Taiwan. Healthcare (Basel).

[8] Chido-Amajuoyi OG, Mantey DS, Clendennen SL, Pérez A (2017). Association of tobacco advertising, promotion and sponsorship (TAPS) exposure and cigarette use among Nigerian adolescents: implications for current practices, products and policies. BMJ Glob Health.

[9] Repositori Badan Kebijakan Pembangunan Kesehatan (2019). Laporan Nasional Riskesdas 2018.

[10] World Health Organization (2019). Lembar Informasi GYTS Indonesia 2019.

[11] Nasional KPP (2015). Rencana Pembangunan Jangka Panjang Nasional 2015-2019.

[12] Database Peraturan. Peraturan Pemerintah (PP) Nomor 109 Tahun 2012 tentang pengamanan bahan yang mengandung zat adiktif berupa produk tembakau bagi kesehatan.

[13] Astuti PAS Policy incoherence and unwillingness of the Indonesian government to curb its alarming tobacco epidemic. Tobacco Control.

[14] Marynak K, Gentzke A, Wang TW, Neff L, King BA (2018). Exposure to electronic cigarette advertising among middle and high school students - United States, 2014-2016. MMWR Morb Mortal Wkly Rep.

[15] Nicksic NE, Brosnan PG, Chowdhury N, Barnes AJ, Cobb CO (2019). ‘Think it. Mix it. Vape it.’: a content analysis on E-Cigarette radio advertisements. Subst Use Misuse.

[16] Fauzi R, Areesantichai C (2020). Factors associated with electronic cigarettes use among adolescents in Jakarta, Indonesia. Journal of Health Research.

[17] Stubbs T (2021). Commercial determinants of youth smoking in ASEAN countries: a narrative review of research investigating the influence of tobacco advertising, promotion, and sponsorship. Tob Induc Dis.

[18] Megatsari H, Damayanti R, Kusuma D (2023). The influence of anti-smoking messages to Indonesian youth smoking behavior: the Indonesian 2019 Global Youth Tobacco Survey (GYTS). BMC Public Health.

[19] CDC (2019). Global Youth Tobacco Survey 2019. Indonesia 2019.

[20] World Health Organization (2003). WHO Framework Convention on Tobacco Control.

[21] Astuti PAS, Freeman B (2018). Tobacco company in Indonesia skirts regulation, uses music concerts and social media for marketing.

[22] Siahaya IA, Smits T (2021). Sport CSR as a hidden marketing strategy? A study of Djarum, an Indonesian tobacco company. Sport Soc.

[23] Soerojo W, Bigwanto M, Susilo D, Wiyono NH, Aditama TY, Achadi A (2020). Fakta Tembakau Indonesia 2020 Data Empirik untuk Pengendalian Tembakau.

[24] Lovato C, Watts A, Stead LF (2011). Impact of tobacco advertising and promotion on increasing adolescent smoking behaviours. Cochrane Database Syst Rev.

[25] BPOM Indonesia (2021). Laporan tahunan 2020 pusat pengembangan pengujian obat dan makanan nasional badan pengawas obat dan makanan.

[26] The Constitutional Court of the Republic of Indonesia Petition on Review of Cigarette Advertising Rejected.

[27] Komisi Perlindungan Anak Indonesia (2017). Tanpa Kompromi, KPAI Minta UU Penyiaran Cantumkan Larangan Iklan Rokok.

[28] Kemp S (2021). Digital 2021: Indonesia.

