# Supplementary material for: Tobacco advertising, promotion, sponsorship and youth smoking behavior: The Indonesian 2019 Global Youth Tobacco Survey (GYTS)
Source: Tob Induc Dis. 2023 Dec 12;21:163. doi: 10.18332/tid/174644 (PMC10714413; doi:10.18332/tid/174644)
Supplement: Supplementary file 1 [file TID-21-163-s1.pdf]

Supplementary Table 1. The Construction of The Three Exposures

No.	Exposure Items	Original questions Indonesian GYTS 2019	Exposure Indicators
1.	Tobacco advertisement exposure	1. During the past 30 days, did you see any people using tobacco on TV, in videos, or in movies? 2. Over the past 30 days, have you seen cigarette advertisements/promotions/brand names/cigarette logos on television? 3. During the past 30 days, did you see any advertisements for cigarette products in newspapers or magazines? 4. During the past 30 days, did you see any cigarette advertisements in outdoor media? (such as billboards, banners, posters, digital billboards) 5. During the past 30 days, did you see any cigarette advertisements when you opened in the internet or social media? 6. During the past 30 days, did you see any advertisements or promotions for cigarette products in sales centers (such as shops, stalls, kiosks and minimarket)?	Respondents answered yes or no to each question. We calculated as no exposure, one exposure and more than one exposure
2.	Tobacco promotion exposure	1. Would you ever use or wear something that has a cigarette company or cigarette product name or picture on it such as a match, t-shirt, hat, or sunglasses? 2. Do you have something (for example, t-shirt, pen, backpack, hat or sun glasses) with a cigarette product brand logo on it? 3. Has a person working for a tobacco company ever offered you a free tobacco product? 4. Have you ever got free cigarettes/discounted cigarette coupons/vouchers from cigarette companies?	Respondents answered yes or no to each question. We calculated as no exposure, one exposure, and more than one exposure

3.	Tobacco sponsorship exposure	1. During the past 30 days, did you see any advertisements for cigarette products when you attended sports events?<br>2. During the past 30 days, did you see any advertisements for cigarette products at music concerts?<br>3. During the past 30 days, did you see any advertisements for cigarette products at community events/social gatherings?	Respondents answered yes or no to each question. We calculated as no exposure, one exposure and more than one exposure

Supplementary Table 2. Respondent distribution, Indonesia 2019 GYTS by current smoker, ever smoker, and cigarette consumption per day

Covariates & Exposures	Current Smoker (n=8,228)					Ever smoker (n=8,579)					Cigarette consumption per day (n=1,470)				
	Non-Smoker		Current Smoker		p-value	No		Yes		p-value	Low		High		p-value
	%	95% CI	%	95% CI		%	95% CI	%	95% CI		%	95% CI	%	95% CI	
**Covariates**															
**Sex**															
Female	97.7	[96.8,98.4]	2.3	[1.6,3.2]	***	88.3	[86.5,89.8]	11.7	[10.2,13.5]	***	78.3	[68.0,86.0]	21.7	[14.0,32.0]	**
Male	61.6	[57.7,65.4]	38.4	[34.6,42.3]		33	[28.4,37.8]	67	[62.2,71.6]		61	[56.2,65.6]	39	[34.4,43.8]	
**Grade**															
Junior high school	81.3	[78.7,83.7]	18.7	[16.3,21.3]	0.88	62.7	[59.2,66.2]	37.3	[33.8,40.8]	0.40	71	[67.1,74.6]	29	[25.4,32.9]	***
Senior high school	81	[78.2,83.6]	19	[16.4,21.8]		60.8	[57.9,63.6]	39.2	[36.4,42.1]		45	[38.6,51.5]	55	[48.5,61.4]	
**Weekly spending money**															
More than IDR50,000	83.1	[80.5,85.5]	16.9	[14.5,19.5]	**	64.9	[61.5,68.2]	35.1	[31.8,38.5]	0.28	52	[43.2,60.8]	48	[39.2,56.8]	**
IDR41,000-IDR50,000	83.5	[79.9,86.5]	16.5	[13.5,20.1]		62.6	[57.6,67.3]	37.4	[32.7,42.4]		57.7	[49.4,65.6]	42.3	[34.4,50.6]	
IDR31,000-IDR40,000	84.8	[80.5,88.3]	15.2	[11.7,19.5]		60.3	[54.3,66.1]	39.7	[33.9,45.7]		61.1	[49.0,72.0]	38.9	[28.0,51.0]	
IDR21,000-IDR30,000	83	[79.6,85.9]	17	[14.1,20.4]		63.6	[59.1,67.9]	36.4	[32.1,40.9]		64.3	[55.7,72.2]	35.7	[27.8,44.3]	
IDR11,000-IDR20,000	79	[76.1,81.5]	21	[18.5,23.9]		60	[56.9,63.0]	40	[37.0,43.1]		65.6	[58.2,72.3]	34.4	[27.7,41.8]	
Less than IDR11,000	78	[75.3,80.6]	22	[19.4,24.7]		60.6	[56.7,64.4]	39.4	[35.6,43.3]		69.7	[64.1,74.9]	30.3	[25.1,35.9]	
Usually don't have any spending money	79.2	[72.8,84.5]	20.8	[15.5,27.2]		60.4	[50.6,69.4]	39.6	[30.6,49.4]		62	[50.2,72.5]	38	[27.5,49.8]	
**Secondhand Smoking**															
People who smoked inside your home in your presence in the past seven days															
0 days	91.7	[90.1,93.0]	8.3	[7.0,9.9]	***	75.5	[72.7,78.2]	24.5	[21.8,27.3]	***	65.6	[58.4,72.2]	34.4	[27.8,41.6]	**
1 to 2 days	74.6	[71.6,77.3]	25.4	[22.7,28.4]		57	[53.7,60.2]	43	[39.8,46.3]		69	[63.4,74.1]	31	[25.9,36.6]	
3 to 4 days	71.6	[67.6,75.3]	28.4	[24.7,32.4]		47.6	[43.3,52.1]	52.4	[47.9,56.7]		60.4	[52.4,67.9]	39.6	[32.1,47.6]	
5 to 6 days	69.9	[61.9,76.8]	30.1	[23.2,38.1]		43.7	[36.6,51.0]	56.3	[49.0,63.4]		59.7	[46.2,71.9]	40.3	[28.1,53.8]	
7 days	71.9	[69.1,74.5]	28.1	[25.5,30.9]		49.5	[46.4,52.6]	50.5	[47.4,53.6]		55.8	[49.2,62.1]	44.2	[37.9,50.8]	
Saw anyone who smoked inside the school building or outside school property															
No	85.6	[83.4,87.5]	14.4	[12.5,16.6]	***	68.9	[65.6,71.9]	31.1	[28.1,34.4]	***	65.1	[59.2,70.6]	34.9	[29.4,40.8]	0.157
Yes	77.7	[75.6,79.6]	22.3	[20.4,24.4]		56.7	[54.1,59.2]	43.3	[40.8,45.9]		60.7	[55.5,65.6]	39.3	[34.4,44.5]	
Do your parents smoke															

Covariates & Exposures	Current Smoker (n=8,228)					Ever smoker (n=8,579)					Cigarette consumption per day (n=1,470)				
	Non-Smoker		Current Smoker		p-value	No		Yes		p-value	Low		High		p-value
No	82.5	[80.4,84.5]	17.5	[15.5,19.6]	*	64.3	[61.0,67.4]	35.7	[32.6,39.0]	**	65.9	[61.0,70.5]	34.1	[29.5,39.0]	**
Yes	79.6	[76.9,82.1]	20.4	[17.9,23.1]		59.4	[56.6,62.1]	40.6	[37.9,43.4]		58.3	[52.7,63.7]	41.7	[36.3,47.3]	
**Noticed anti-tobacco advertising**															
None	90.9	[88.3,92.9]	9.1	[7.1,11.7]	***	72.2	[68.0,76.0]	27.8	[24.0,32.0]	***	57.2	[47.2,66.6]	42.8	[33.4,52.8]	0.27
One	85.3	[83.5,86.9]	14.7	[13.1,16.5]		68.7	[66.2,71.1]	31.3	[28.9,33.8]		60	[52.6,67.0]	40	[33.0,47.4]	
More than one	74.6	[71.9,77.2]	25.4	[22.8,28.1]		53.3	[50.2,56.5]	46.7	[43.5,49.8]		63.9	[59.4,68.2]	36.1	[31.8,40.6]	
**Attitude towards Smoking Behaviour**															
Positive	92.2	[91.0,93.3]	7.8	[6.7,9.0]	***	72.6	[70.2,74.8]	27.4	[25.2,29.8]	***	85.8	[81.7,89.1]	14.2	[10.9,18.3]	***
Negative	59.9	[56.7,62.9]	40.1	[37.1,43.3]		42.2	[38.9,45.6]	57.8	[54.4,61.1]		53.5	[48.4,58.5]	46.5	[41.5,51.6]	
**Exposures**															
**Tobacco Advertisement exposure**															
None	89.9	[87.2,92.1]	10.1	[7.9,12.8]	***	73.2	[69.2,76.9]	26.8	[23.1,30.8]	***	82.3	[69.3,90.5]	17.7	[9.5,30.7]	**
One	84.3	[81.5,86.7]	15.7	[13.3,18.5]		66	[62.4,69.5]	34	[30.5,37.6]		64.2	[54.0,73.2]	35.8	[26.8,46.0]	
More than one	79.7	[77.5,81.6]	20.3	[18.4,22.5]		60.1	[57.4,62.7]	39.9	[37.3,42.6]		60.7	[56.2,65.1]	39.3	[34.9,43.8]	
**Tobacco promotion exposure**															
None	87.5	[85.9,88.9]	12.5	[11.1,14.1]	***	70	[67.7,72.2]	30	[27.8,32.3]	***	68.2	[62.4,73.6]	31.8	[26.4,37.6]	***
One	74.9	[71.5,78.1]	25.1	[21.9,28.5]		49.6	[45.6,53.6]	50.4	[46.4,54.4]		64	[57.9,69.8]	36	[30.2,42.1]	
More than one	60	[55.6,64.2]	40	[35.8,44.4]		41.7	[37.5,46.1]	58.3	[53.9,62.5]		52.8	[46.5,58.9]	47.2	[41.1,53.5]	
**Tobacco sponsorship exposure**															
None	87.3	[86.0,88.6]	12.7	[11.4,14.0]	***	68.2	[65.8,70.5]	31.8	[29.5,34.2]	***	67	[61.5,72.0]	33	[28.0,38.5]	***
One	67.3	[63.6,70.9]	32.7	[29.1,36.4]		48.5	[44.6,52.4]	51.5	[47.6,55.4]		53.1	[46.7,59.4]	46.9	[40.6,53.3]	
More than one	59.6	[54.8,64.3]	40.4	[35.7,45.2]		41.4	[36.4,46.6]	58.6	[53.4,63.6]		61.9	[55.8,67.7]	38.1	[32.3,44.2]	

*** p<0.001, ** p<0.01, * p<0.05

Supplementary Figure 1. Bivariate analysis

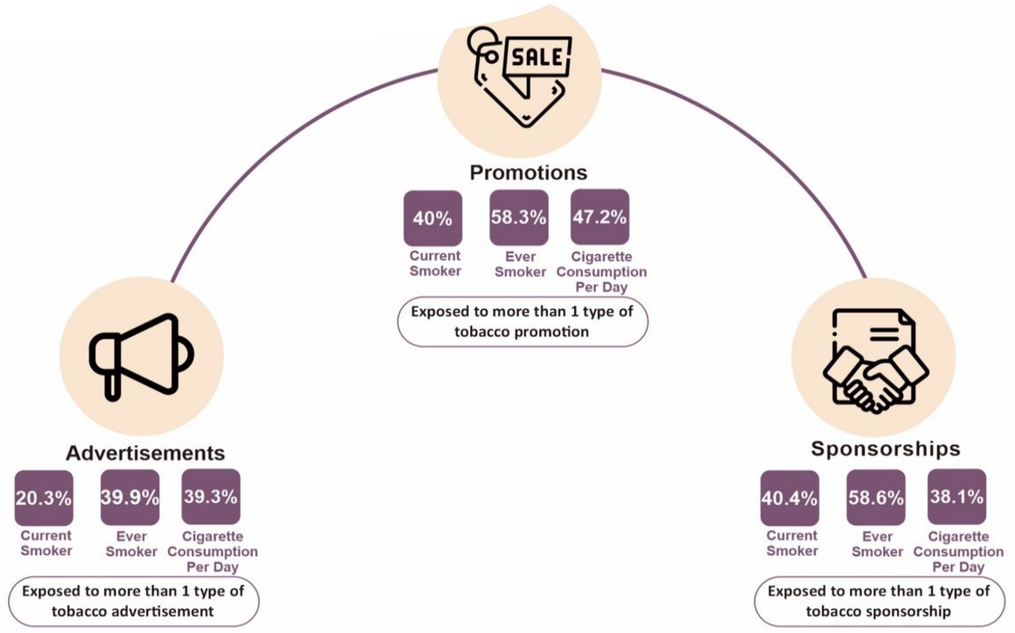

Supplementary Figure 2. Multivariable analysis

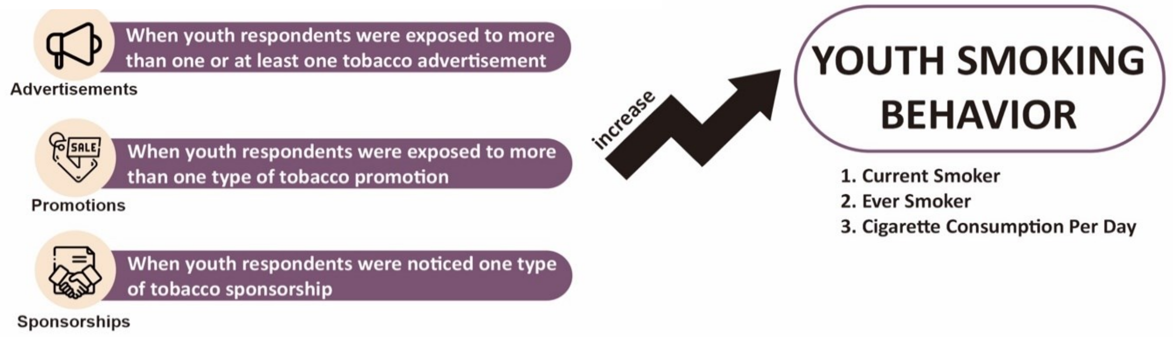

© 2023 Megatsari H. et al.
